# NAVIGATING THE PATHWAY TO CARE IN ADULTS WITH CEREBRAL PALSY

**DOI:** 10.1093/geroni/igac059.518

**Published:** 2022-12-20

**Authors:** Heidi Haapala

**Affiliations:** University of Michigan, Ann Arbor, Michigan, United States

## Abstract

As individuals with CP age, they face unique challenges which complicate their ability to access and receive appropriate health care. These problems exist at the level of the health care system, the clinician, and the individual. At the system level, there is an inadequate number of professionals who are informed of and interested in the care of adults with CP. Adult caregivers are often not knowledgeable about and may feel less competent about patients with CP. Differences in the physiologic development of individuals with CP render well-established clinical protocols for risk screening of chronic diseases less effective. Dr. Haapala runs an adult CP clinic and will present her clinical experience with treating complex patients with CP. She will present specific information pertaining to her work in studying and treating chronic overlapping pain in adults with CP, as well as her efforts to reduce polypharmacy and opioid dependence in her patients.

